# Desmoplastic Melanoma: Report of 5 Cases

**DOI:** 10.1155/2009/679010

**Published:** 2009-11-30

**Authors:** A. M. Manganoni, C. Farisoglio, S. Bassissi, D. Braga, F. Facchetti, M. Ungari, P. G. Calzavara-Pinton

**Affiliations:** ^1^Department of Dermatology, University Hospital Spedali Civili, 25123 Brescia, Italy; ^2^Department of Dermatology, Casa di Cura Poliambulanza, 25123 Brescia, Italy; ^3^Department of Pathology I, University Hospital Spedali Civili, 25123 Brescia, Italy

## Abstract

*Background.* The clinical presentation of desmoplastic melanoma is often challenging. We report the experience of the Melanoma Unit of Spedali Civili University Hospital of Brescia, Italy. *Method.* Study subjects were drawn from 1770 patients with histologica confirmed melanoma. Within this group, desmoplastic melanoma developed in 5 patients. For each diagnosed melanoma, histological characteristics, treatment, and outcomes were evaluated. *Results.* Of the 5 patients described in this study, 2 were males and 3 females. The average age was 62.4 years ranging from 56 to 68 years. Breslow thickness ranged from 2.1 to 12 mm with a mean thickness of 5.8 mm. Primary treatment of 5 patients included a wide local excision of their primary lesions. *Conclusions.* Desmoplastic melanoma is a rare neoplasm which clinically may mimic other tumours or cutaneous infiltrate of uncertain significance. The diagnosis is hiastopathological and radical resection is necessary.

## 1. Background

Desmoplastic melanoma (DM) is a rare variant of malignant melanoma first described by Conley et al. in 1971 as an invasive melanoma composed of spindle cells surrounded by abundant collagen [1]. The clinical presentation of desmoplastic melanoma is often challenging. Usually it has an innocuous clinical appearance and it is described as an indurated discoid papule, plaque, or nodule. It most commonly arises in chronically sun-damaged skin. Rarely, DM may present on a mucosal surface. We describe our experience at Melanoma Unit of Spedali Civili University Hospital in Brescia, Italy, with patients whose melanoma was reported to be desmoplastic.

## 2. Material and Methods

Study subjects were drawn retrospectively from 1770 patients with histological confirmed melanoma, including melanoma in situ, between January 1, 1984, and January 1, 2009. These patients were followed up by the Melanoma Unit of Spedali Civili University Hospital in Brescia, Italy. All the patients gave an informed consent to be entered into the data-base. A total of 5 patients (0.3%) who had been given a diagnosis of cutaneous desmoplastic melanoma, which was histologically confirmed, were identified (Table 1). All patients were staged with the use of the American Joint Committee on Cancer (AJCC) staging classification [2]. The patient's sex, ethnic origin, age, and Fitzpatrick skin types at diagnosis were recorded. For each diagnosed melanoma, histologic classification, anatomic location, tumor thickness (Breslow method), Clark's anatomical level of invasion, ulceration, regression, neurotropism, lymphatic invasion and number of mitoses per square millimetre were evaluated. Melanomas were classified as being located in the head and neck, trunk (including chest, back, abdomen, and buttock), upper extremity (including arm, forearm, and hand), and lower extremity (including thigh, leg, and foot). Treatment and outcomes were also examined.

## 3. Results

In the period between January 1, 1984, and January 1, 2009 5 patients with cutaneous desmoplastic melanoma were identified. The incidence of desmoplastic melanoma cases in our population was 0.3% (5/1770). Of the 5 patients evaluated, 2 were males and 3 females. All patients were Caucasians. The average age was 62.4 years ranging from 56 to 68 years. Of the 5 documented patients, percentages of Fitzpatrick skin types were as follows: II 60% (3 pz) and III 40% (2 pz). Primary melanomas were found in different body sites: 1 (20%) on the trunk, 2 (40%) on the upper extremity, and 2 (40%) on the lower extremity. None of our 5 cases had a documented family history of melanoma. Breslow thickness ranged from 2.1 to 12 mm with a mean thickness of 5.8 mm. One patient had a lesion with neurotropism (case 5). Primary treatment of 5 patients included wide and deep local excision of their primary lesions. 4 out of 5 patients underwent sentinel lymph node biopsy (SLNB) and no patient was found to have a positive sentinel node. Follow-up ranged from 2 to 113 months, with an average of 49 months, and during follow-up period any patient had disease progression or developed local recurrence.

## 4. Conclusions

**Figure 1:**
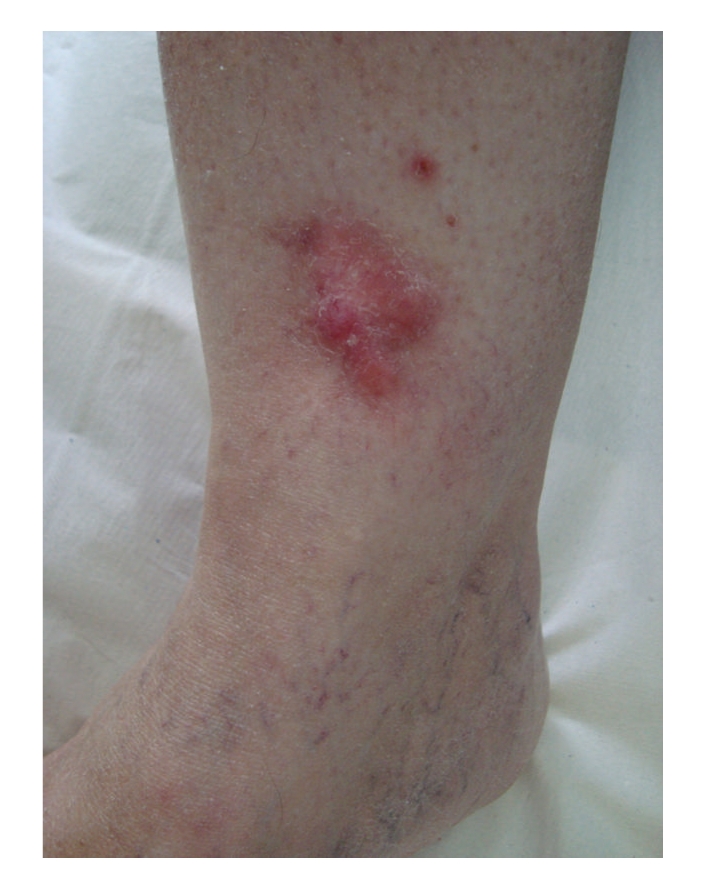
Clinical appearance of desmoplastic melanoma (patient n°4): red plaque of 5 × 3 cm on left leg. The diagnoses that are suspected clinically range from basal cell carcinoma to dermatofibrosarcoma protuberans or amelanotic melanoma.

The incidence of desmoplastic melanoma cases in our population was 0.3% (5/1770). Of the 5 patients evaluated, 2 (40%) were males and 3 (60%) female. This female predominance in sex distribution is not in accordance with previous case series [3]. The median age at diagnosis of DM is approximately 10 years higher than the one for conventional melanoma [4]. In our case the average age was 62.4 years ranging from 56 to 68 years. Head and neck is the preferred site for DM; in the series from the Massachusetts General Hospital, 75% of the tumors occurred in this anatomic site [5]. None of our 5 cases had a primary melanoma in the head and neck. The clinical presentation of desmoplastic melanoma is often challenging. Usually it has an innocuous clinical appearance and it is described as an indurated discoid papule, plaque, or nodule. Pigmentation is frequently absent (Figure 1), although a lentigo or lentigo maligna-like discoloration adjacent to the nodule is not uncommon [6]. The diagnoses that are suspected clinically range from benign (scar, dermatofibroma, melanocytic nevus) to malignant (basal cell or squamous cell carcinoma, sarcoma, or amelanotic melanoma) lesions. In a series of 113 cases of desmoplastic melanoma melanoma was the initial clinical diagosis in only 27% of the cases [3].

**Figure 2:**
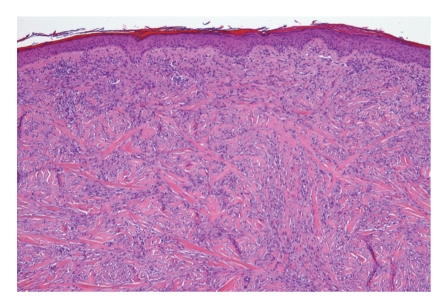
Desmoplastic melanoma: atypical spindle cells in a dense fibous matrix (haematoxylin and eosin; original magnification ×10).

**Figure 3:**
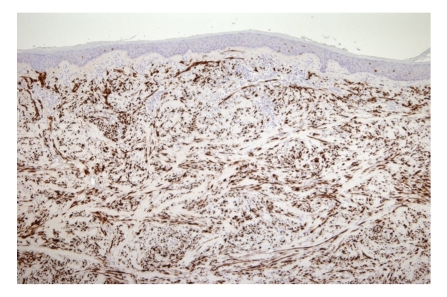
Desmoplastic melanoma: S-100 immunoreactivity in the dermal spindle cells (original magnification ×10).

There are no dermoscopic criteria for this rare neoplasm probably because dermoscopic examination is not routinely performed before excising a lesion that is often clinically not considered as melanocytic. Debarbieux et al. reported the dermoscopic features for six cases of desmoplastic melanoma. In this short series only half of the cases exhibited one classical feature of a melanocytic lesion while other cases were recognized on the basis of the presence of figures of regression (all six) such as white scar-like and “peppering”, multiple (>4) colours (five out of six), and melanoma-related vascular patterns (five out of six) such as linear-irregular vessels and milky-red areas [7]. The diagnosis of DM is histopathological; the histology of classic DM (Figures 2 and 3) is defined as a dermal-based, paucicellular proliferation of atypical spindle cells in a sclerotic or neuromatous stroma with evidence of melanocytic differentiation [8]. There is histological subclassification of DM into two variants: pure desmoplastic melanoma (pDM) when desmoplasia is prominent throughout the tumor and mixed desmoplastic melanoma (mDM) when desmoplasia constitutes only a part of an otherwise nondesmoplastic invasive melanoma.

The majority of DMs are larger than 1 mm in thickness at the time of diagnosis, and many tumors measure more than 4 mm. This probably results from the difficulties in clinical diagnosis. In our series, Breslow thickness ranged from 2.1 to 12 mm with a mean thickness of 5.8 mm. The role of SLNB in the DM is not defined as well. Several studies have shown that patients with melanomas of desmoplastic type have a lower frequency of sentinel node positivity than patients with nondesmoplastic melanomas. In view of this, some authors have advocated avoiding SLNB in patients with desmoplastic melanoma [9–11]. 4 out of 5 patients underwent sentinel lymph node biopsy and no patient was found to have a positive sentinel node. However, we think that it is important in the stage of the disease when Breslow is equal or larger than 1 mm. Assessment of nodal involvement allows indentifying patients at risk for locoregional recurrence. Literature on DM reports an incidence of ‘‘local recurrence” higher than the one of conventional melanoma in patients with DM [1, 12–15]. However, comparison of local recurrence rates between DM and conventional melanomas is problematic. In most series of recurrent melanoma, no attempt is made to precisely define local recurrence and distinguish between persistent melanoma and cutaneous metastases [16]. With our patients, follow-up ranged from 2 to 113 months, with an average of 49 months, and during follow-up period any patient had disease progression or developed local recurrence. DM is a rare neoplasm which clinically may mimic other tumours or cutaneous infiltrate of uncertain significance. Clinical features, in fact, might be similar to melanoma but could also be quite different. The diagnosis is hiastopathological and radical resection is necessary. Current controversies regarding locoregional treatment strategies warrant further investigation.

## Figures and Tables

**Table 1:** Clinical features of 5 patients with cutaneous desmoplastic melanoma.

Patient no.	Age (y)/Sex	Fitzpatrick skin types	Site	Breslow depth (mm)	SLNB	Neurotropis m	Follw-up (mos)/status
1	65/F	II	Upper extremity	2.4	—	absent	113/Alive
2	65/F	III	Lower extremity	2.5	negative	absent	68/Alive
3	56/M	II	Upper extremity	2.1	negative	absent	56/Alive
4	58/F	II	Lower extremity	12	negative	absent	5/Alive
5	68/M	III	Trunk	10	negative	present	2/Alive

SLNB sentinel lymph node biopsy.

## References

[1] Conley J, Lattes R, Orr W (1971). Desmoplastic malignant melanoma (a rare variant of spindle cell melanoma). *Cancer*.

[2] Balch CM, Sober AJ, Soong SJ, Gershenwald JE (2003). AJCC melanoma staging committee. The new melanoma staging system. *Seminars in Cutaneous Medicine and Surgery*.

[3] de Almeida LS, Requena L, Rütten A (2008). Desmoplastic malignant melanoma: a clinicopathologic analysis of 113 cases. *American Journal of Dermatopathology*.

[4] Hawkins WG, Busam KJ, Ben-Porat L (2005). Desmoplastic melanoma: a pathologically and clinically distinct form of cutaneous melanoma. *Annals of Surgical Oncology*.

[5] Carlson JA, Dickersin GR, Sober AJ (1995). Desmoplastic neurotropic melanoma: a clinicopathologic analysis of 28 cases. *Cancer*.

[6] Payne WG, Kearney R, Wells K (2001). Desmoplastic melanoma. *American Surgeon*.

[7] Debarbieux S, Ronger-Salve S, Dalle S, Balme B, Thomas L (2008). Dermoscopy of desmoplastic melanoma: report of six cases. *British Journal of Dermatology*.

[8] George E, McClain SE, Slingluff CL, Polissar NL, Patterson JW (2009). Subclassification of desmoplastic melanoma: pure and mixed variants have significantly different capacities for lymph node metastasis. *Journal of Cutaneous Pathology*.

[9] Gyorki DE, Busam K, Panageas K, Brady MS, Coit DG (2003). Sentinel lymph node biopsy for patients with cutaneous desmoplastic melanoma. *Annals of Surgical Oncology*.

[10] Livestro DP, Muzikansky A, Kaine EM (2005). Biology of desmoplastic melanoma: a case-control comparison with other melanomas. *Journal of Clinical Oncology*.

[11] Pawlik TM, Ross MI, Prieto VG (2006). Assessment of the role of sentinel lymph node biopsy for primary cutaneous desmoplastic melanoma. *Cancer*.

[12] Smithers BM, McLeod GR, Little JH (1992). Desmoplastic melanoma: patterns of recurrence. *World Journal of Surgery*.

[13] McCarthy SW, Scolyer RA, Palmer AA (2004). Desmoplastic melanoma: a diagnostic trap for the unwary. *Pathology*.

[14] Bruijn JA, Mihm MC, Barnhill RL (1992). Desmoplastic melanoma. *Histopathology*.

[15] Heenan PJ (2004). Local recurrence of melanoma. *Pathology*.

[16] Busam KJ (2005). Cutaneous desmoplastic melanoma. *Advances in Anatomic Pathology*.

